# Implications of COVID-19 in high burden countries for HIV/TB: A systematic review of evidence

**DOI:** 10.1186/s12879-020-05450-4

**Published:** 2020-10-09

**Authors:** Jacques L. Tamuzi, Birhanu T. Ayele, Constance S. Shumba, Olatunji O. Adetokunboh, Jeannine Uwimana-Nicol, Zelalem T. Haile, Joseph Inugu, Peter S. Nyasulu

**Affiliations:** 1 Division of Epidemiology and Biostatistics, Faculty of Medicine and Health Sciences, Stellenbosch University, Cape Town, South Africa; 2 Department of Population Health, Aga Khan University, Nairobi, Kenya; 3 School of Nursing and Midwifery, Aga Khan University, Nairobi, Kenya; 4 DSI-NRF Centre of Excellence in Epidemiological Modelling and Analysis, Stellenbosch University, Stellenbosch, South Africa; 5 School of Public Health, College of Medicine and Health Science, University of Rwanda, Kigali, Rwanda; 6 Department of Social Medicine, Ohio University, Heritage College of Osteopathic Medicine, Dublin, USA; 7 Departments of Public Health, School of Health Sciences, Central Michigan University, Mount Pleasant, USA; 8 Division of Epidemiology, School of Public Health, Faculty of Health Sciences, University of the Witwatersrand, Johannesburg, South Africa

**Keywords:** COVID-19, SARS-CoV, MERS-CoV, SARS-CoV-2, HIV, TB, Co-infection

## Abstract

**Background:**

The triple burden of COVID-19, tuberculosis and human immunodeficiency virus is one of the major global health challenges of the twenty-first century. In high burden HIV/TB countries, the spread of COVID-19 among people living with HIV is a well-founded concern. A thorough understanding of HIV/TB and COVID-19 pandemics is important as the three diseases interact. This may clarify HIV/TB/COVID-19 as a newly related field. However, several gaps remain in the knowledge of the burden of COVID-19 on patients with TB and HIV. This study was conducted to review different studies on SARS-CoV, MERS-CoV or COVID-19 associated with HIV/TB co-infection or only TB, to understand the interactions between HIV, TB and COVID-19 and its implications on the burden of the COVID-19 among HIV/TB co-infected or TB patients, screening algorithm and clinical management.

**Methods:**

We conducted an electronic search of potentially eligible studies published in English in the Cochrane Controlled Register of Trials, PubMed, Medrxiv, Google scholar and Clinical Trials Registry databases. We included case studies, case series and observational studies published between January, 2002 and July, 2020 in which SARS-CoV, MERS-CoV and COVID-19 co-infected to HIV/TB or TB in adults. We screened titles, abstracts and full articles for eligibility. Descriptive and meta-analysis were done and results have been presented in graphs and tables.

**Results:**

After removing 95 duplicates, 58 out of 437 articles were assessed for eligibility, of which 14 studies were included for descriptive analysis and seven studies were included in the meta-analysis. Compared to the descriptive analysis, the meta-analysis showed strong evidence that current TB exposure was high-risk COVID-19 group (OR 1.67, 95% CI 1.06–2.65, *P* = 0.03). The pooled of COVID-19/TB severity rate increased from OR 4.50 (95% CI 1.12–18.10, *P* = 0.03), the recovery rate was high among COVID-19 compared to COVID-19/TB irrespective of HIV status (OR 2.23, 95% CI 1.83–2.74, *P* < 0.001) and the mortality was reduced among non-TB group (*P* < 0.001).

**Conclusion:**

In summary, TB was a risk factor for COVID-19 both in terms of severity and mortality irrespective of HIV status. Structured diagnostic algorithms and clinical management are suggested to improve COVID-19/HIV/TB or COVID-19/TB co-infections outcomes.

## Background

The triple burden of COVID-19, tuberculosis (TB) and human immunodeficiency virus (HIV) is one of the major and persistent global health challenges of the twenty-first century. In the last two decades, three major coronavirus epidemics have been reported worldwide. Those epidemics are caused by different agents: severe acute respiratory syndrome coronavirus (SARS-CoV) in 2002, Middle East respiratory syndrome coronavirus (MERS-CoV) in 2012 and the current of SARS-CoV-2 outbreak, known as COVID-19 [1]. In 2002, SARS-CoV originated in Guangdong province, China, spreading to 37 countries, and the subsequent global epidemic was associated with 8096 cases and 774 deaths [2]. Ten years later, the MERS-CoV spread to 27 countries, causing 2494 infected cases and 858 deaths worldwide [2, 3]. The novel coronavirus currently known as (2019-nCoV) was identified in 2019 and is the third highly pathogenic CoV detected, with a fatality rate varying across countries and ranges of age. In addition, the 2019-nCoV transmissibility is higher, the 2019-nCoV mean R_0_ (R_0_ is used to measure virus transmissibility) ranged from 3.3 to 5.5, and it appeared higher than that of SARS-CoV (2–5) and MERS-CoV (2.7–3.9) [2–6]. An estimated 18,142,718 people have been infected and 691,013 have died from December 2019 to 04 August 2020, yielding a fatality rate of 3.81% % worldwide [7].

HIV, TB and newly Emerging Infectious Diseases such as Coronavirus epidemics are expected to overlap in high HIV and TB burden countries. The intersecting coronavirus, HIV and TB epidemics in countries with a high burden of HIV and TB infections pose several public health challenges. In fact, TB is the leading immune-suppressing infection and the most common cause of death among HIV-infected patients [8]. Worldwide, there were 37.9 million [32.7 million–44.0 million] people living with HIV and 1.7 million [1.4 million–2.3 million] people became newly infected with HIV at the end of 2018 [9]. WHO reports that people living with HIV are 20 times more likely to develop TB than their counterparts [10]. It is estimated that 1.1 million people worldwide live with TB and HIV, 80% of whom live in sub-Saharan Africa [11]. Since the emergence of HIV, TB incidence is increasing and causing a high mortality rate among people living with HIV/AIDS over the last ten years [12]. In the post-mortem, the overall prevalence of TB in adults and children was huge and accounted for almost 40% of HIV-related facility-based deaths in adults in resource-limited countries [13]. This is greater than the WHO/UNAIDS estimate that overall TB accounts for approximately 25% of HIV/AIDS related deaths worldwide [13]. How COVID-19 will manifest itself in persons co-infected with HIV/TB is still uncertain [14]. Populations infected with HIV and TB, those with undiagnosed pulmonary TB (PTB), drug-resistant tuberculosis or complex presentations such as disseminated types and those who have only started PTB treatment may be at elevated risk for severe responses if they are infected with COVID-19 [14]. In the future, lung lesions associated with COVID-19 may increase the risk of PTB, which induces a true vicious circle of HIV-TB-COVID-19 co-infection. TB incidence is also anticipated to increase in high burden HIV/TB countries including sub-Saharan countries with high COVID-19 burden. While COVID-19 continues to spread across the world, many areas face the risk of infection with SARS-CoV-2 and the obstacles and challenges to sustaining the continuum of HIV and TB treatment in high-burden HIV/TB countries are increasing [14]. Co-infection SARS-CoV/HIV/TB was previously not a major threats because SARS-CoV and MERS-CoV pandemics did not occur in countries with high HIV/TB burden. Since December 2019, COVID-19 is spreading very fast, with the high HIV/TB burden countries not spared from the pandemic and the number of new COVID-19 cases is expected to rise in the next few months. The intersecting coronavirus, TB and HIV epidemics in sub-Saharan African countries where HIV and TB have the highest prevalence and incidence respectively, pose many challenges from the point of view of COVID-19/TB diagnostics, COVID-19/HIV/TB clinical management and post COVID-19 epidemic TB incidence as COVID-19 pulmonary fibrosis may rapidly increase TB incidence [15].

**Fig. 1:**
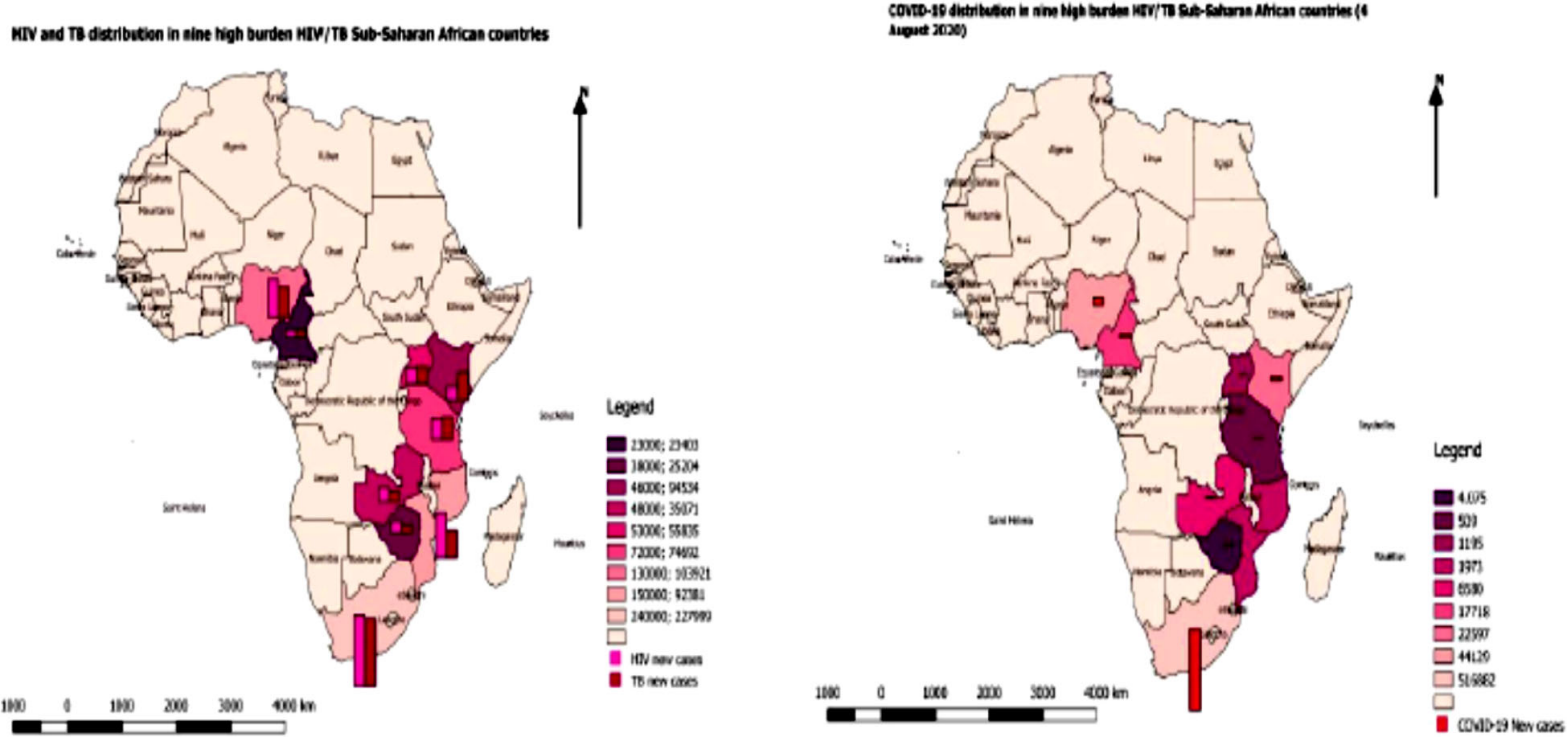
Distribution of COVID-19, HIV and TB in the nine high burden countries in Sub-Saharan African

In fact, the pathogenicity of COVID-19 could be accelerated in people living with HIV who have compromised immunity [1]. Recent evidence has indicated a substantial association between coronavirus-related Lower Respiratory Tract Infections (LRTIs) and increased risk of death in immune-compromised individuals [16, 17]. At the same time, the depletion of CD4 T cells in HIV and latent TB-infection disrupts the integrity and architecture of TB granulomas in the lung, thus facilitating progression to active TB [18–20]. Similarly, TB promotes a microenvironment that facilitates the replication of HIV-1 via various mediators [21]. In fact, irreversible improvements in lung architecture after SARS-CoV and/or TB play a significant role in both SARS-CoV and TB pathogenesis. Nonetheless, severe SARS-CoV can induce the development of rapid pulmonary fibrosis compared with mild courses of SARS-CoV disease usually advanced to organize phase diffuse alveolar damage (DAD) and eventual long-term deposition of fibrous tissue [15]. On the whole, SARS-CoV, HIV and TB co-infection may have deleterious consequences in all stages of SARS, HIV and TB because the triple pandemics are related in the immuno-pathological phase, constituting a vicious circle. A thorough understanding of the interactions between the three deadly pandemics is crucial. Reviewing the statistics in relation to high burden HIV/TB countries and recent World Health Organization data on COVID-19 in Sub-Saharan Africa; the following countries may expect an increased number of TB during or post COVID-19: South Africa, Nigeria, Cameroon, Kenya, Tanzania, Mozambique, Zambia, Zimbabwe and Uganda. The distribution of estimated new HIV cases (2018), new TB cases and relapses (2018) and COVID-19 cases (04 August 2020) are respectively 240,000; 227,999; 516,882 (South Africa), 130,000; 103,921; 44,129 (Nigeria), 23,000; 23,403; 17,718 (Cameroon), 46,000; 94,534; 22,597 (Kenya), 72,000; 74,692; 509 (Tanzania), 150,000; 92,381; 1973 (Mozambique), 48,000; 35,071; 6580 (Zambia), 38,000; 25,204; 4075 (Zimbabwe) and 53,000; 55,835; 1195(Uganda) [7, 10, 22]. The map was drawn to illustrate the distribution of COVID-19, HIV and TB in the nine high burden countries in Sub-Saharan African (Fig. 1). The aim of this study was to review different studies on SARS-CoV or MERS-CoV associated with HIV/TB co-infection or TB only and understands the interactions between HIV, TB and COVID-19 and its implications on the burden of the COVID-19 among TB/HIV patients, screening algorithm and management.

## Methods

The protocol was accepted by the international prospective register of systematic reviews (PROSPERO) (identification number: CRD42020181457). We conducted a systematic review of the literature to examine SARS-CoV or MERS-CoV associated with HIV/TB or TB co-infection. As we anticipated heterogeneity in the literature, meta-analysis and descriptive analysis were undertaken. Meta-analysis was based on random-effects modeling using Review Manager 5.3 [23] and Meta-Essentials [24] was also used to compute Egger’s regression and Begg and Mazumdar rank correlation test to evaluate possible publication bias. The odds ratio (OR) for COVID-19/HIV co-infection or COVID-19 in relation to TB exposure was used as the summary measure of risk in these meta-analyses. Heterogeneity across included studies was estimated by *χ*^2^ and *I*^2^. Forest plots and relevant supporting statistics were examined. Meta-analyses for subgroups (on the basis of COVID-19/HIV/TB vs COVID-19/TB) were also undertaken to investigate heterogeneity between the subsets. We also computed the test of two proportions with STATA version 14 to compare SARS, MERS or COVID-19 disease severity compared to TB and/or HIV in descriptive analysis.

We utilized formal methods of literature search, selection of articles for inclusion, an abstraction of data and quality assessment, and synthesis of results to review the literature on to examine SARS-CoV or MERS-CoV or COVID-19 associated with HIV/TB or TB co-infection.

### Inclusion criteria

The inclusion criteria were studies published in English, from January 2020 until July 2020 that established co-occurrence of SARS-CoV, MERS-CoV, COVID-19 HIV and TB. Study designs included case reports, case series and observational studies (case-control, prospective and retrospective cohorts). Studies reporting COVID-19/HIV co-infection without screening PTB, those reporting other outcomes, letters to the editor, theoretical and incomplete studies were excluded. The outcomes include TB occurrence (before, during or after SARS, MERS or COVID-19), SARS, MERS or COVID-19 severity (mild, moderate, severe and critical stages) in case of HIV/TB or TB co-infections, the mean time of COVID-19 severe/critical stages occurrence and the recovery and mortality rates.

### Search strategy

We searched eligible studies from 01 January 2002 to 27 July 2020 through Medline (PubMed), Google Scholar, Medrxiv and the Cochrane Library without any study design, published in English. Additionally, the WHO COVID-19 database [22] and Clinicaltrials.gov were also used to search for ongoing and completed studies related to co-infection COVID-19/HIV/TB. The following terms were used “SARS-CoV”, “MERS-CoV”, “COVID-19”, “SARS-CoV-2” AND “pulmonary tuberculosis”, “PTB”, “lung TB”, “TB” AND “HIV/TB co-infection” AND “TB/SARS co-infection” AND “TB/MERS co-infection” “TB/Covid-19 co-infection” AND “HIV/SARS co-infection” AND “HIV/MERS co-infection” AND “HIV/COVID-19 co-infection”. Relevant articles published in English that resulted from the searches, and references cited therein, were reviewed and duplicate studies were removed. After removing duplicates, we checked the title and abstract and reviewed full-text, inclusions and exclusions were recorded following PRISMA guidelines presented in the form of a PRISMA flow diagram and detailed reasons recorded for exclusion. Critical appraisal checklists appropriate to each study design were applied and conducted in pairs (JTL and PSN).

### Data extraction

A customized data extraction form was designed and piloted prior to data extraction. For each study included, we collected the following information: authors and publication year, title and journal, study country, study design, sample size, participants characteristics such as age and sex, the number of conditions included (SARS-CoV, MERS-CoV, COVID-19, HIV and TB) and the outcomes include TB occurrence (before, during or after SARS, MERS or COVID-19), SARS, MERS or COVID-19 severity (mild, moderate, severe and critical stages) in case of HIV/TB or TB co-infections, the average time of COVID-19 severe/critical stages occurrence, and the recovery and mortality rates. The data extraction was conducted in pair by (JLT and BTA). Conflict resolution was conducted by a third co-author (PSN).

### Assessment of study quality

Two reviewers (JTL and BTA) independently assessed study quality based on the Newcastle-Ottawa scale (NOS) [25]. The Newcastle-Ottawa scale assessed the selection, comparability and exposure of a case-control study and selection, comparability, and outcome of a cohort study. Nine stars reflect maximum ranking, and the sample with over 6 stars was considered to be of reasonably high quality. Any questions about the content of the included studies were determined in consultation with another reviewer (PSN).

## Results

### Search results

**Fig. 2:**
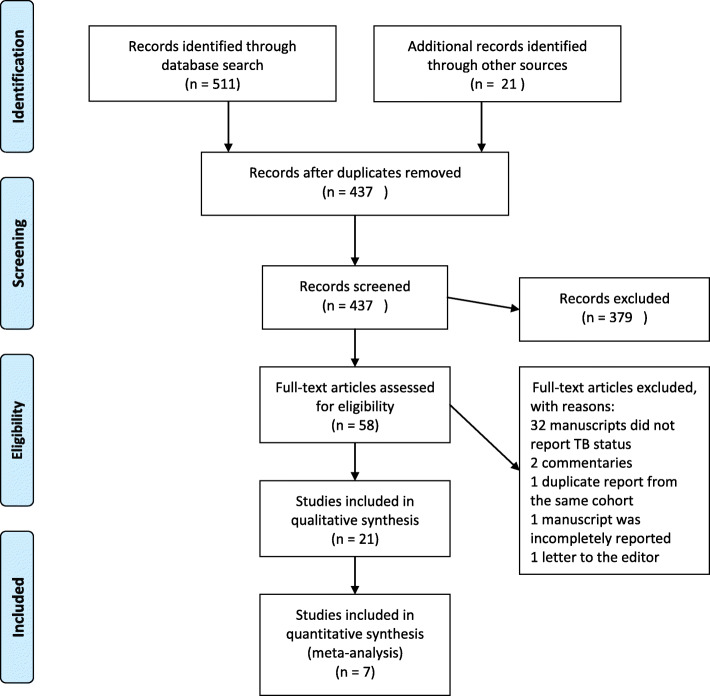
Flow diagram of SARS-CoV or MERS-CoV or COVID-19 associated with TB/HIV or TB studies included in the review. Note. From PRISMA: www.prisma-statement.org

Electronic search identified 532 articles. Inclusions and exclusions were reported following PRISMA guidelines presented in the form of a PRISMA flow diagram (Fig. 2) with reasons for exclusion recorded (Table 1) as follows: 95 duplicates were removed; after reading the titles of articles, 379 articles were removed. Among 58 records screened, 21 full-text studies were assessed for eligibility. Thirty-seven articles were excluded because there were either incomplete or irrelevant articles. Twenty-one studies were included for qualitative analysis, of which five were case reports, eight case series, one case-control and seven cohort studies (Table 2). Seven out of eight observational studies were included in the meta-analysis. One retrospective cohort was included in the descriptive analysis because COVID-19/TB co-infected cases were identified in both cases and exposed groups [47]. Each article that met selection criteria was fundamentally assessed for Author/Country, Population/Study design, Exposures, Comparators, Treatments, TB occurrence and SARS/MERS/COVID-19 severity, recovery and mortality rate.

**Table 1:** Description of studies excluded in review

N	Author/Country	Population/Study design	Reasons for exclusion
1	Shalhoub 2015 Saudi Arabia	A patient with MERS-CoV/HIV co-infection/case study	TB status was not reported
2	Bogorodckaya 2020 Russia	three TB patients co-infected with COVID-19/ case study	Cases were incompletely reported.
3	Wang 2020 China [26]	A patient with COVID-19/HIV co-infection/case report	TB status was not reported
4	Zhu 2020 China	A patient with COVID-19/HIV co-infection/case report	TB status was not reported
5	Zhao 2020 China	A patient with COVID-19/HIV/HCV co-infection/ case report	TB status was not reported
6	Baluku 2020 Uganda	A patient with COVID-19/HIV co-infection /case report	TB status was not reported
7	Blanco 2020 Spain	Five cases of COVID-19/HIV co-infection/ clinical case series	None reported TB status
8	Riva 2020 Italy	Three cases with COVID-19/HIV co-infection / case series	None reported TB status
9	Aydin 2020 Turkey	Three patients with COVID-19/HIV co-infection /case series	Outcomes of interest were not reported
10	Benkovic 2020 USA	Four patients with COVID-19/HIV Co-infection/Case series	Outcomes of interest were not reported
11	Haddad 2020 USA	A case with COVID-19/HIV co-infection/Case report	TB screening was not reported
12	Gervasoni 2020 Italy	47 COVID-19/HIV co-infected patients Retrospective study	Outcomes of interest were not reported
13	Wang 2020 China [26]	A patient with COVID-19/HIV Co-infection/Case report	TB status was not reported
14	Härter 2020 Germany	33 COVID-19/HIV co-infected patients Retrospective study	Outcomes of interest were not reported
15	Wu 2020 China	Two patients with COVID-19/HIV co-infection/Case series	TB screening was not reported
16	Del 2020 Spain [27]	77,590 COVID/HIV co-infected cases	Outcomes of interest were not reported
17	Bulled 2020 South Africa	Comment on COVID-19/HIV/TB co-infection	This was a commentary
18	Tadolini 2020 Italy	49 patients with COVID-19/TB co-infection	Duplicate report from the same cohort
19	Chen 2020 China [28]	COVID-19/TB burden	Letter to the editor without case report
20	Drain 2020 USA	Explanatory article on COVID/HIV burden	TB screening was not reported
21	Karim 2020 USA	Included percentage of COVID-19/HIV/TB report	No primary data reported
22	Pang 2020 China	Included COVID-19/TB cases	A correspondence
23	Wang 2020 China [26]	A case of COVID-19/HIV co-infection	The outcomes of interest were not reported
24	Kilds 2020 USA	A case of COVID-19/HIV co-infection	The outcomes of interest were not reported
25	Ridgway 2020 USA	A case series of five COVID-19/HIV co-infection	Had no outcome of interest reported
26	Sigel 2020 USA	Eighty eight COVID-19/HIV co-infection	Had no outcome of interest reported
27	Patel 2020 USA	A case study of COVID-19/HIV co-infection	The interest outcome was not reported
28	Guo 2020 China	A survey among COVID-19/HIV co-infected cases	The interest outcome was not reported
29	Shalev 2020 USA	31 patients with COVID-19/HIV co-infection	The outcomes of interest were not reported
30	Karmen-Tuohy 2020 USA	A case control of 21 patients COVID-19/HIV co-infection	The outcomes of interest were not reported
31	Toombs 2020 United Kingdom	Three Cases with COVID-19/HIV co-infection	Do not contain the outcome of interest
32	Ruan 2020 China	Three cases with COVID-19/HIV co-infection	Had no outcome of interest reported
33	Sun 2020 Singapore	A case of SARS-2/HIV co-infection	Had no outcome of interest reported
34	Richardson 2020 USA	43 cases of SARS-2/HIV co-infection	Do not contain the outcome of interest
35	Ho 2020 USA	93 cases of SARS-s/HIV co-infection	The outcomes of interest were not reported
36	Su 2020 China	Two cases with SARS-2/AIDS co-infection	The outcomes of interest were not reported
37	Kumar India	A case of COVID-19/HIV co-infection	Had no outcome of interest reported

**Table 2:** Description of studies included in review

Author/Country	Population/Study design	Exposures	Comparators	Treatments	TB occurrence	SARS-CoV/MERS/COVID-19 Severity rate/Time	SARS-CoV/MERS-CoV/COVID-19 Recovery rate/Time	Mortality rate
Liu 2006 China [29]	-Three males of 48(case 1), 18 (case 2) and 20 years (case 3) old with confirmed SARS-CoV -case series	PTB SARS-CoV	N/A	Corticosteroids mechanical ventilation Anti TB drugs	PTB diagnosed while case 1 was in the hospital. Case 2 and 3 were known TB on treatment.	Two patients developed mild SARS-CoV and one developed severe stage.	All of them recovered form SARS-CoV and continue anti-TB drugs	N/A
Low 2004 Singapore [30]	-Two males of 54 and 39 years old with confirmed SARS-CoV -Case series	PTB SARS-CoV	N/A	intravenous immunoglobulin short course of high-dose corticosteroids Anti TB drugs	PTB developed after four and 2 months prior to SARS-CoV.	Both of them developed severe SARS-CoV	Both of the cases recovered from SARS-CoV	N/A
Wong 2004 Hong Kong [31]	-30 years old male with confirmed SARS-CoV and HIV on ART -Case report	HIV Latent TB SARS-CoV	N/A	Abacavir 300 mg Efavirenz 600 mg Kaletra Tenofovir 300 mg Ribavirin 1200 mg prednisolone 25 mg	Diagnosed with PTB during hospitalization	Mild course SARS	The case recovered from SARS-CoV	N/A
Alfaraj 2017 Kingdom of Saudi Arabia [32]	−13-year-old girl and A 30-year-old female with confirmed MERS-CoV -Case series	PTB MERS-CoV	N/A	intensive care admission Anti TB drugs	Both patients initially had TB before MERS-CoV	The 13 years old had severe MERS-CoV. However, the disease severity was moderate with the 30 years old.	Both of the cases recovered from MERS-CoV	N/A
Singh 2020 India [33]	76-year- old female with confirmed COVID-19 Case study	PTB COVID-19	N/A	hydroxychloroquine 400 mg twice daily in addition to antibiotics. Anti TB drugs	Diagnosed with TB during admission	Mild to moderate COVID-19	The patient recovered from COVID-19	N/A
He 2020 China [34]	All three patients were males with 26, 67 and 76 years -Case series	Previous TB PTB COVID-19	N/A	Lopinavir + Ritonavir Arbidol Methyl prednisolone Antibiotics Traditional Chinese medicine Intravenous immunoglobulin Ventilatory support Antituberculosis	Past medical history of TB years ago for all the three patients	All of them had severe type of COVID-19 10 days after onset for the first symptom	All of the three cases recovered from COVID-19	N/A
Cutler 2020 USA [35]	61-year-old male with confirmed COVID-19 and history of Parkinson’s disease. Case study	PTB COVID-19	N/A	Hydroxychloroquine Oxygen supplementation Anti-TB drugs	TB diagnosed in hospitalization	Critical COVID-19 stage	Recovered for COVID-19	N/A
Çınar 2020 Turkey [36]	55-year-old male with a history of myelodysplastic Syndrome, immunocompromised, kidney failure and confirmed with COVID-19 Case study	Disseminated TB COVID-19	N/A	COVID-19 convalescent plasma Favipiravir meropenem Ventilatory support Anti-TB drugs	TB diagnosed in hospitalization	Critical COVID-19 stage	Recovered for COVID-19	N/A
Faqihi 2020 Saudi Arabia [37]	60 year-old male, hypertensive and diabetic, with confirmed COVID-19 Case study	PTB COVID-19	N/A	Lopinavir/ritonavir Ribavirin Dexamethasone Prophylactic anticoagulation Supportive ICU care Anti-TB drugs	Previous TB history	Critical COVID-19 stage	Recovered for COVID-19 after 20 days	N/A
Liu 2020a China [38]	48 year-old, 26-year-old and 46-year-old males with confirmed COVID-19/TB co-infection Case series	PTB COVID-19	N/A	Arbidol Moxifloxacin Linezolid immunomodulatory therapy with thymopentin Ventilatory support Anti-TB drugs	The first and the last case had LTBI and the second was a previous MDR-TB	All of them developed severe/critical COVID-19 courses	All of them recovered from COVID-19 with the range of 9 to 14 days	N/A
Tham 2020 Singapore [39]	32-year-old, 33-year-old, 22-year old and 40-year old males COVID-19/TB co-infected patients Case series	PTB COVID-19	N/A	COVID-19 antiviral drugs Anti-TB drugs	N/A	N/A	All of them recovered.	N/A
Stochino 2020 Italy [40]	20 confirmed cases of COVID-19/TB co-infection. Among them, one case was also HIV-infected. The median age was 39 years Case series	PTB Disseminated TB COVID-19	N/A	Hydroxychloroquine. Ventilatory support Anti-TB drugs	N/A	7 out of 20 developed severe/critical COVID-19 13 out of 20 developed mild/moderate COVID-19 Average of 32 [range 14–57] days	19 out of 20 patients recovered.	1 patient died.
Chen 2020 China [28]	Only four cases of COVID-19/TB co-infection were included in a cohort of 203 confirmed COVID-19 cases. Case series	PTB COVID-19	Survivors vs deceased	N/A	N/A	N/A	N/A	1 case out of 4 died.
Du 2020 China [41]	8 confirmed cases of COVID-19/TB prospective cohort study	PTB COVID-19	Survivors vs deceased	N/A	N/A	N/A	N/A	COVID-19/TB: 0/8 COVID-19: 21/171
Davies 2020 South Africa [42]	3978 COVID-19/HIV co-infected patients 18,330 COVID-19 infected patients, public sector patients aged ≥20 years. Population prospective cohort study	Previous TB status Latent TB HIV COVID-19	COVID-19/HIV/TB vs COVID-19/TB	Antiretroviral therapy Anti-TB COVID-19 antiviral therapies	COVID-19/HIV/TB current TB: 172/3863 Previous TB: 864/3863 COVID-19/TB current TB: 145/ 17,820 Previous TB: 10/510	N/A	COVID-19/HIV/TB: 1039/1094 COVID-19: 2827/2884 COVID-19/TB: 979/1034 COVID-19: 16841/17296	COVID-19/HIV/TB Current TB: 16/172 Previous TB: 42/864 COVID-19/TB Current TB: 10/145 Previous TB: 45/834 COVID-19/HIV/TB: 58/1034 COVID-19/HIV: 57/2884 COVID-19/TB: 55/1034 COVID-19: 455/17296
Karla 2020 Philipines [43]	The matched sample 4510 consisted of COVID-19 patients, of which 113 had confirmed TB. The mean age of the total sample was 48.9 years Longitudinal matched cohort analysis	Confirmed TB, which was defined as a history of or a current diagnosis of TB.	COVID-19/TB co-infection vs COVID-19/no TB	N/A	N/A	Admitted in the hospital COVID-19/TB: 67/106 COVID-19: 236/424	COVID-19/TB Recovered: 57 /106 COVID-19 Recovered: 302/424	COVID-19/TB Died: 25 /106 COVID-19 Died: 46/ 424
Liu 2020 China [38]	−36 confirmed COVID-19 cases Among which 13 were IGRA+ve to TB, mean age: 47 years Case-control study	Previous TB status Latent TB COVID-19	Case series study of 115 bacterial and 62 other viral pneumonia. Controls selected in the same setting.	N/A	Previous TB: 8/13 Current TB: 3/13	Severity: Mild/Moderate: 0/27 Severe/Critical: 3/9 -severe/critical COVID-19: 3.4 days after initial symptom development.	N/A	N/A
Li 2020 China [2]	A total of 549 patients with COVID-19 were enrolled. The median age of study population was 60 years. Retrospective study	PTB COVID-19	Non-Severe vs severe COVID-19	N/A	N/A	Severe: 4/269 Not severe: 5/ 279	N/A	N/A
Zhang 2020 China [44]	−140 confirmed COVID-19 cases, 2 of whom had secondary PTB -Retrospective study	PTB COVID-19	Non-severe vs Severe COVID-19	N/A	N/A	Severity COVID-19/TB Severe: 2/58 Not severe: 0/82	N/A	N/A
Zhang 2020b China [44]	1350 confirmed cases of COVID-19 among which 8 were COVID-19/TB co-infected cases. Age (44.1 ± 17.9) years Retrospective cohort study	PTB COVID-19	Non-severe vs Severe COVID-19	N/A	N/A	Severe: 3/229 Not severe: 2/1121	N/A	N/A
Motta 2020 Italy, Belgium, Brazil, France, Russia, Singapore, Spain, Switzerland [47]	- 49 confirmed TB and COVID-19 cases, median age 70 years Retrospective cohort study	Previous TB status Latent TB COVID-19 One patient with HIV	20 confirmed COVID-19/TB cases Retrospective cohort study (Cohort B)	Hydroxychloroquine Lopinavir/ ritonavir Azythromycin Empiric antibiotic Enoxaparine 4000 IU Dexamethasone Oxygen through non-rebreather, 15lt/min Anti TB drugs	−3 with previous TB diagnosed −8 (simultaneous diagnosis of COVID-10 and TB −11 had COVID-19 diagnosed between 7 and 75 days) after the TB diagnosis	−5 patients had severe COVID-19 − 8 patients developed critical COVID-19 -Critical COVID-19: median of 9 (range 6–14) days after COVID-19 diagnosis	60 cases recovered from COVID-19/TB co-infection the both cohorts.	9 out of 69 died from COVID-19/TB co-infection

Table 2 presents a summary of 21 included studies. The review included 28,387 COVID-19, 6 SARS-CoV and 2 MERS-CoV participants with HIV/TB or TB. Among them, 1294 were COVID-19/TB, 1094 COVID-19/HIV/TB, 5 SARS-CoV/TB, 2 MERS-CoV/TB and 1 SARS-CoV/HIV/TB. Four cohort studies [41, 44, 45, 48], one case control study [38] and four case series [28, 29, 34, 49] were conducted in China, two case series [30, 39] were done in Singapore, one case series [32] and one case control study [37] were undertaken in Saudi Arabia, a case series [40] was conducted in Italy, one cohort study was done in South Africa [42] and another in the Philippines [43], one case study was conducted in the United State of America [35], other three case studies were found in Turkey [36], Hong Kong [31] and India [33] respectively. Lastly, a retrospective cohort was undertaken in eight countries (Italy, Belgium, Brazil, France, Russia, Singapore, Spain, and Switzerland) [47].

### Quality assessment of included studies

The methodological validity of included studies for determining the consistency of case-control study and cohort studies in meta-analyses was based on the NOS [25]. This method explores three major components: range, comparability and exposure. The NOS uses a star chart with ratings from 0 to 9 for case–control and cohort studies. Since the requirements for a study’s high or low quality are not well known, we considered a study with a higher score than the six of each form of study to be a high-quality study. Among included studies, two scored seven and high, three scored six and the other two studies scored less than six and were therefore considered low quality. The NOS scores for the included studies are shown in Table 1 (Supplementary material).

### Descriptive analysis

Fourteen studies (one observational study, eight case series and five case studies) were included in the descriptive analysis. We identified 113 cases with SARS-CoV, MERS-CoV or COVID-19 associated to HIV/TB or TB. The computed median age between case studies and case series was 32 years compared to the cohort study median age of 70 years [47]. Males had higher SARS-CoV or MERS-CoV or COVID-19 associated to HIV/TB or TB co-infections than females with 70% (28/40). Table 2 describes all cases. For further clarifications, cases were grouped as follows:

#### Cases of SARS-CoV, MERS-CoV or COVID-19 with previous history of PTB diagnosis

Six studies (an observational study, four case series and one case study) [29, 32, 34, 37, 47, 49] included cases known to have a history of PTB (sputum smear–negative for acid-fast bacilli) and became infected with SARS-CoV (two cases) or MERS-CoV (two cases) or COVID-19 (eight cases). PTB diagnosis was made based on previous exposure to TB, relevant symptoms of typical PTB, chest radiographs suggestive of active disease or IGRA (Interferon Gamma Release Assay). SARS-CoV or MERS-CoV was confirmed based on amplification of SARS-CoV/MERS-CoV RNA by reverse transcriptase–polymerase chain reaction (RT-PCR) from sputum. SARS-CoV/TB co-infected cases were managed with corticosteroids and anti TB drugs. Clinical management was not specified for MERS-CoV/TB co-infected cases; however anti TB drugs were administered. Lopinavir/r, Arbidol, Ribavirin, corticoids (dexamethasone and methyl prednisolone), prophylactic anticoagulation, empirical antibiotics, traditional Chinese medicine and anti-TB drugs were indicated to COVID-19/TB co-infected cases. Eight out of fourteen had severe/critical disease course among which one case of SARS-CoV, one of MERS-CoV and six cases of COVID-19 and had a long recovery process.

#### SARS-CoV or COVID-19 cases with PTB co-infection

Seven studies (an observational study, two case series and four case studies) [29, 31, 33, 35, 36, 47, 49] diagnosed PTB (positive acid-fast bacilli smear on sputum samples or IGRA), while cases were admitted for SARS-CoV (two cases) and COVID-19 (twelve cases). SARS-CoV and COVID-19 were confirmed by RT-PCR, and microscopy as well as medical imaging diagnosed TB. A case of SARS-CoV/HIV/TB co-infection [31] was managed with abacavir/efavirenz/kaletra/tenofovir/ribavirin, prednisolone and anti TB drugs and another case with SARS-CoV/TB co-infection [29] was managed with mechanical ventilation, corticosteroids and anti TB drugs. All COVID-19/TB co-infected cases were managed in the same way as for previous PTB/COVID-19 cases.

#### Cases of PTB with history of SARS-CoV

Two cases were diagnosed with PTB with positive bacilli smear respectively four and two months after SARS-CoV [30]. At day 80 of disease on convalescence, one of the cases was positive for coronavirus IgG serum antibody and the other case was positive for SARS coronavirus by PCR of an endotracheal tube test, as well as coronavirus IgM and IgG antibodies in the blood. Both of them had severe COVID-19 before developing PTB. Intravenous immunoglobulin and a short course of high-dose corticosteroids were indicated during the SARS course and anti TB drugs were administered during the TB course. The said patients remained clinically stable at follow-up.

Cases were stratified by PTB diagnosis. 41.36% (12/29) of cases of SARS-CoV, MERS-CoV or COVID-19 had previous history of PTB diagnosis. 74% of SARS-CoV or COVID-19 Cases had PTB co-infection, and 6.89% (2/29) of PTB had a history of SARS-CoV. The test of two proportions between ‘severe/critical SARS, MERS and COVID-19 cases with HIV/TB or TB co-infection 53% (20/38)’ versus ‘mild/moderate SARS, MERS and COVID-19 cases with HIV/TB or TB co-infection 47% (18/38)’ was not statistically significant (*P* = 0. 6009). The onset of COVID-19 severe/critical stages was mean of 3.4 days [38] and a median of 9 days [47] for two observational studies and 10 days for a case series [34]. Three studies [28, 30, 32, 37, 39, 40, 47] reported the recovery and mortality rate among SARS, MERS and COVID-19 cases with HIV/TB or TB co-infection which were respectively 90.26% (102/113) and 9.74% (11/113). The mortality rate of 9.74% among COVID-19/TB or COVID/HIV/TB co-infected patients should be considered with caution because of poor study design and small sample size. However, the mortality rates for COVID-19/TB or COVID/HIV/TB co-infection seem to be higher than the mortality rate of 3.81% for COVID-19 worldwide [7]. Three studies [37, 38, 40] reported COVID-19 recovery time from 9 to 54 days. This is important to highlight that qualitative analysis included three cases related to SARS-CoV/HIV/TB co-infection (one case) [31] and COVID-19/HIV/TB co-infection (two cases) [28–30, 32, 34, 37–41, 44, 45, 47–49]. Among them, SARS-CoV/HIV/TB co-infected case developed mild disease course, one COVID-19/HIV/TB developed severe COVID-19 and the last case died.

### Meta-analysis

We included seven observational studies (six cohort studies and one case control study) in the meta-analysis (Fig. 2). A case control and four cohort studies were conducted in China [38, 41, 44, 45, 48] and two other cohort studies were undertaken in South Africa [42] and the Philippines [43] (see Table 2). One cohort included COVID-19/HIV/TB and COVID-19/TB co-infected groups were included in the TB occurrence outcome, with a total sample of 2015 participants. COVID-19 severity included three cohort studies and one case control for which the total sample size was 2074 participants. A total of 22,838 and 23,017 participants were included in recovery and mortality rates respectively. Each of them included two and three cohort studies respectively. The results of the meta-analysis based on seven observational studies including HIV/TB or TB as exposures that may impact on COVID-19 outcomes were described as follows:

### TB occurrence

**Fig. 3:**
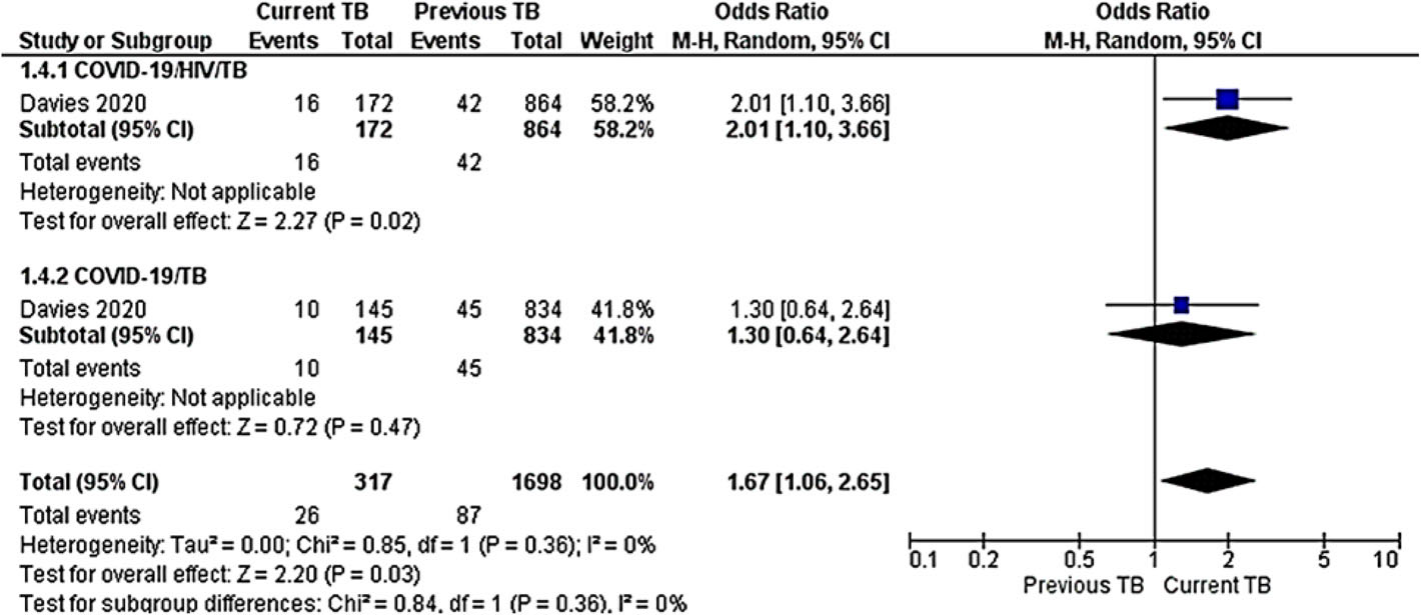
Meta-analysis of TB occurrence among COVID-19/HIV/TB or COVID-19/TB co-infections. Outcome: TB occurrence

This included previous and current TB occurrence among COVID-19/HIV/TB and COVID-19/TB co-infections. Only one study included TB occurrence [42]. Current TB showed a strong risk of COVID-19 among HIV-infected cases OR 2.01 (95% CI 1.10–3.66), *P* = 0.02 compared to uninfected HIV cases OR 1.30 (95% CI 0.64–2.64), *P* = 0.47. TB occurrence pooled results between subgroup COVID-19/HIV/TB and COVID-19/TB was OR 1.67(95%CI 1.06–2.65, *P* = 0.03). The test for subgroup differences was not statistically significant with *I*^2^ = 0%, *P* = 0.36 (Fig. 3).

### COVID-19 severity

**Fig. 4:**
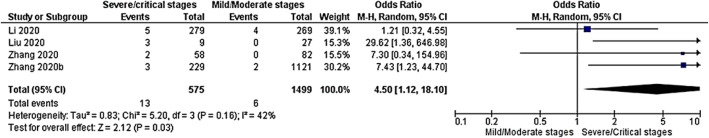
Meta-analysis of COVID-19 severity among COVID-19/TB co-infected patients. Outcome: COVID-19 severity

Three cohort studies [44, 45, 48] and one case control studies [38] were included to compare mild/moderate versus severe/critical COVID-19 stages in COVID-19/TB co-infected patients. The pooled result revealed that the COVID-19/TB group was at high risk of developing severe/critical COVID-19 compared to the COVID-19 group OR 4.50 (95%CI 1.12–18.10, *P* = 0.03). The test of heterogeneity was not statistically significant *P* = 0.16, *I*^2^ = 42% (Fig. 4).

### Recovery rate

**Fig. 5:**
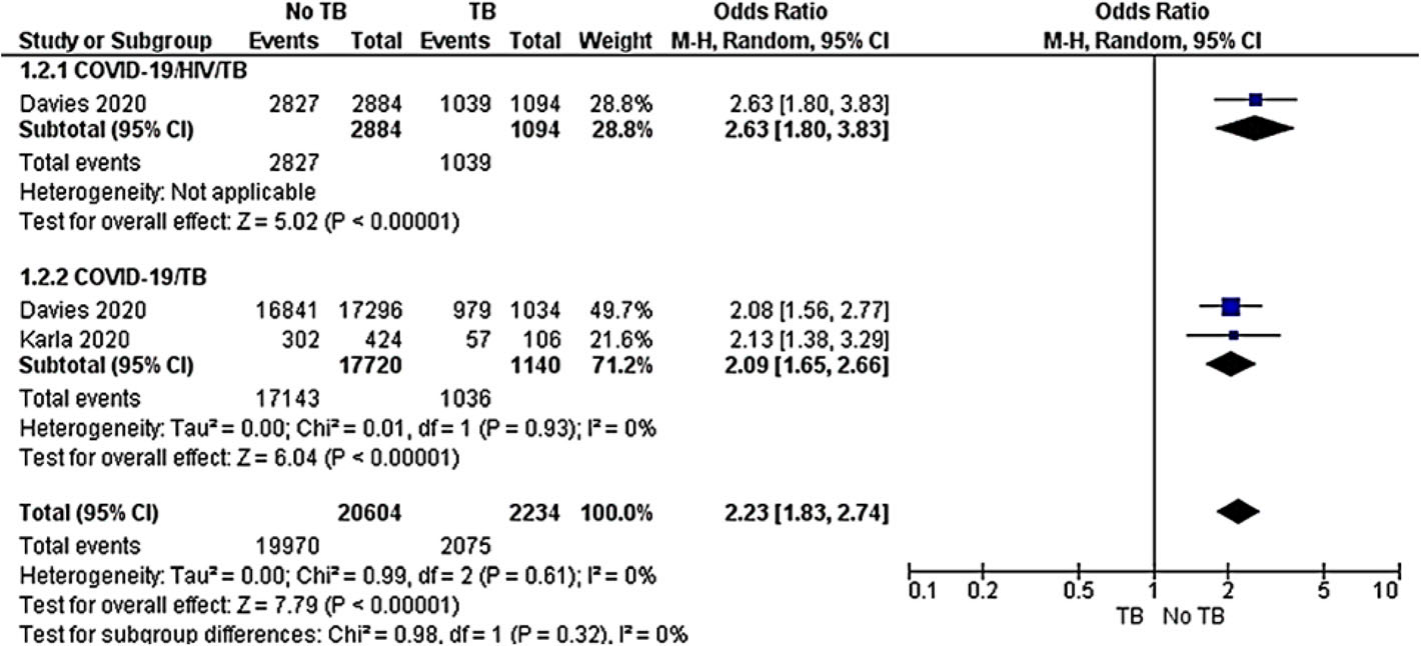
Meta-analysis of COVID-19/HIV/TB versus COVID-19/TB co-infections. Outcome: recovery rate

Two cohort studies [42, 43] were included to evaluate the recovery rate among the COVID-19/HIV/TB group compared to the COVID-19/TB group. The subgroup analysis was performed across the two groups. The COVID-19/HIV/TB subset showed that COVID-19/HIV co-infected group reached the highest odds in recovery rate compared to the COVID-19/HIV/TB co-infected group OR 2.63 (95%CI, 1.80–3.83, *P* < 0.00001). Similarly, the COVID-19 group had the strongest odds of recovering compared to COVID-19/TB co-infected group OR 2.09 (95%CI 1.65–2.66, *P* < 0.00001). The overall result showed that non-TB groups yielded an OR of 2.23 (95%CI 1.83–2.74, *P* < 0.00001) compared to TB in both COVID-19/HIV and COVID-19 groups. The test for subgroup differences was not statistically significant with *P* = 0.32 and *I*^2^ = 0% (Fig. 5).

### Mortality rate

**Fig. 6:**
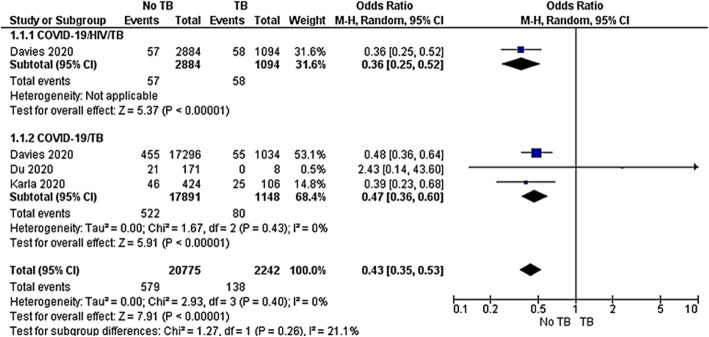
Meta-analysis of TB mortality among COVID-19/HIV/TB or COVID-19/TB co-infections. Outcome: TB mortality

Three observational studies [41–43] compared the mortality rate among COVID-19/HIV/TB and COVID-19/TB co-infected groups. Subgroup analysis was undertaken to evaluate the heterogeneity between the two groups. Among those studies, Davies 2020 included both COVID-19/HIV/TB and COVID-19/TB co-infected cases. The first subgroup revealed that COVID-19/TB co-infected group had a 74% risk reduction of dying compared to the COVID-19/HIVTB co-infected group (OR 0.36, 95%CI 0.25–0.52, *P* < 0.00001). In the same way, the second subgroup analysis including two observational studies showed that the COVID-19 group had a 53% risk reduction of dying compared to the COVID-19/TB co-infected group (OR 0.36, 95%CI 0.36–0.60). The pooled results between non TB and TB in both subgroups revealed OR 0.43, 95%CI 0.35–0.53, *P* < 0.00001. The test for subgroup difference showed no significant heterogeneity across included studies *P* = 0.26, *I*^2^ = 21.1% (Fig. 6).

## Discussion

Reviewing descriptive analysis compared to meta-analysis, meta-analysis illustrated that TB exposure is a COVID-19 risk factor in point of fact TB occurrence, COVID-19 severity, and recovery and mortality rates. However, the descriptive analysis showed the interactions between SARS-CoV, HIV and TB may occur during SARS-CoV or after SARS-CoV. Men are more vulnerable to SARS or MERS or COVID-19 associated to HIV/TB or TB. It is highly likely that both previous SARS-CoV with newly diagnosed PTB acquired active PTB after contracting SARS. This is so because both had laboratory-confirmed clinical syndromes associated with SARS, and both recovered well without anti-TB treatment, with initial biochemical and radiological improvement [29]. The descriptive analysis of cases also found that SARS-CoV could induce a transient suppression of cellular immunity that further predisposed patients to exacerbated reactivation or new TB infection, as is the case with HIV. SARS-CoV and HIV may decrease conjunctly CD4 count and lymphocytes, adding high corticosteroids [29] as a treatment for SARS-CoV may be TB precipitant factors [29]. Following this, SARS-CoV or COVID-19 patients may-be more susceptible to active and latent TB as proven by different studies [29, 31, 33, 35, 36, 47]. It is important to realize that lung lesions due to SARS and/or TB may increase significantly the likelihood of SARS-CoV and TB. Lastly, the descriptive analysis showed COVID-19 time-to-recovery in COVID-19/TB co-infected cases may be longer and severe/critical COVID-19 symptoms may be precocious. An observational study has showed a statistically significant result in time-to-recovery (*P* = 0.0046) [43].

A meta-analysis assessing TB occurrence COVID-19/HIV/TB versus COVID-19/TB co-infected cases demonstrated that the risk of COVID-19 was high among current TB/HIV co-infected cases in subgroup analysis. HIV-infected people are more vulnerable to COVID-19. Although we estimated the pooled COVID-19 rate and the result showed that the COVID-19/TB group was at high risk of developing severe/critical COVID-19 compared to the COVID-19 group. This needs careful interpretation due to a wide overall 95% CI as well as due to studies with different designs. This evidence is supported by two large cohort studies conducted in Spain and South Africa, showing that the risks for PCR-confirmed COVID-19 diagnosis, hospitalization, Intensive Care Unit (ICU) admission, and death among HIV-positive persons receiving ART were greater in men compared to old age [27, 31, 33, 35, 36, 42, 43]. However, the risk for hospitalization varied by the nucleoside transcriptase inhibitor (NRTI) regimen and was lower in patients receiving TDF/FTC versus those receiving other regimens [27, 31, 33, 35, 36, 42, 43].

Our results suggest that the recovery rates between COVID-19/HIV/TB and COVID-19/TB groups were quite the same OR 2.63 (95%CI, 1.80–3.83, *P* < 0.00001) and OR 2.09 (95%CI 1.65–2.66, *P* < 0.00001) respectively. However, COVID-19 patients recovered faster than both COVID-19/HIV/TB and OR 2.09 (95%CI 1.65–2.66, *P* < 0.00001). This observation is supported by qualitative evidence as shown above.

The poor outcome in mortality rate among COVID-19/HIV/TB co-infection compared to COVID-19/TB infection is illustrated in Fig. 6. Both those with COVID-19/HIV/TB and COVID-19/TB co-infection had increased mortality risk compared to COVID-19 participants. Given recent developments that have shown the vulnerability of those ages 18–49 to COVID-19 [50, 51], younger people living with HIV (PLWH) may also be at heightened risk for mortality due to COVID-19 complications. Such risk is predicated on the fact that PLWH under the age of 50 years are both less likely to be diagnosed (and in effect more likely to be immunocompromised) and also less likely to access and be retained in care, yielding viral suppression of a mere 37% for those age 25–34 years [51, 52].

Our review had a number of important limitations, the most important being that almost all included studies were observational and the number of included studies was limited. Nevertheless, heterogeneity was not statistically significant between studies in different subgroup analyses and the Egger regression and Begg and Mazumdar’s test for rank correlation were not statistically significant with *P* = 0.684 and 1.00 respectively, showing that publication bias was minimized.

As shown above, COVID-19/HIV/TB or COVID-19/TB co-infections are a new medical field that needs further attention and research in high burden HIV/TB countries more specifically in sub-Saharan Africa as the co-existence of those three pandemics may imply vulnerability to COVID-19 infections and increase TB occurrence. Clear diagnostic algorithms, exploration of drug–drug interactions and clinical management should be addressed to improve COVID-19/HIV/TB outcomes.

## Review implications: TB, HIV and COVID-19 diagnostics and clinical management

Even though data are scarce, the analysis indicated that COVID-19/HIV/TB or COVID-19/TB co-infections may have poor treatment outcomes. This may be worsened in case TB is not diagnosed and treated early. Furthermore, COVID-19 can shadow TB in HIV-infected people or vice versa. For this reason, we suggest screening for both COVID-19 and TB in HIV-infected people with COVID-19/TB symptoms during the COVID-19 pandemic in countries with high HIV/TB burden. HIV/COVID-19 co-infection requires a simple algorithm and management to boost TB outcomes.

### TB diagnosis in COVID-19/HIV co-infection

Suspected cases of COVID-19 and TB show similar fever and/or respiratory symptoms (difficult respiration, coughing, chest pain, etc.). COVID-19 RT-PCR should be done in real-time for differential diagnosis of cases with unknown respiratory syndromes such as PTB [53]. Due to poor outcomes among COVID-19/HIV/TB or COVID-19/TB co-infections, we recommend COVID-19 real-time RT-PCR should be coupled with Xpert MTB/RIF assay. In suspected HIV/TB co-infected patients, Xpert MTB/RIF should be used first rather than traditional microscopy, culture and drug susceptibility testing (DST) [54]. Instead of collecting upper respiratory tract specimens, lower respiratory tract specimens, such as sputum, bronchoalveolar lavage, and tracheal aspirates should be collected in suspected COVID-19/HIV/TB or COVID-19/TB co-infected patients. COVID-19 real-time RT-PCR may last at least 24 h. At the same time, the Xpert MTB / RIF assay detects *M. tuberculosis* and rifampicin resistance within less than two hours [55]. Xpert MTB/RIF is also a major advance in the diagnosis of TB, particularly for multidrug-resistant (MDR) TB and HIV-associated TB [54]. The Xpert MTB/RIF assay’s sensitivity to detect TB is superior to that of microscopy and comparable to that of solid culture, along with high specificity [55].

This is important to emphasize that possible causes of false negative COVID-19 real-time RT-PCR results in COVID-19/HIV co-infection may be identified in patients on protease inhibitors (PIs) based regimens. We also recommend systematic TB screening in COVID-19/HIV co-infection. The adapted algorithms to diagnose TB in confirmed COVID-19/HIV co-infected adults in high burden HIV/TB countries are described below:

Option 1: This algorithm includes an interrogatory about cough of any duration, fever, short breathing, sore throat, loss of weight, loss of appetite, nausea, hemoptysis and night sweat. Past medical history includes previously confirmed TB, previous TB contact, TB preventive therapies, unsuppressed HIV viral load and CD4 count ≤350 cells/μL. Xpert MTB/RIF assay should be indicated. If Xpert MTB/RIF assay is positive, start anti TB drugs.

Option 2: This algorithm includes symptoms and medical history of COVID-19, HIV and TB as described in option 1. Xpert MTB/RIF assay should be indicated. If Xpert MTB/RIF assay is negative, the culture associated with the chest X-ray should be requested. If abnormal chest X-ray suggestive of TB, start anti-TB drugs, in the meantime while waiting for culture results.

Option 3: This algorithm includes symptoms and medical history of COVID-19, HIV and TB as described in option 1. If Xpert MTB/RIF assay is negative and the X-ray is not suggestive of TB, the culture associated with an approved interferon-gamma release assays (IGRAs) should be performed. Current evidence indicates that IGRAs perform similarly to the tuberculin skin test (TST) at identifying HIV-infected individuals with TB [56]. However, the decision to use either test should be based on country guidelines and resource and logistical considerations. If IGRAs is positive and the culture is negative, start TB preventive treatment. Isoniazid monotherapy for six (6) months is recommended for the treatment of LTBI in both in high burden HIV/TB countries [57]. Rifampicin or rifapentine plus isoniazid daily for three (3) months should be offered as an alternative to six (6) months of isoniazid. However, rifampicin and rifapentine should be prescribed with caution in HIV/COVID-19 co-infection due to potential drug-drug interactions.

Option 4: This algorithm includes a history of previous COVID-19, previous contact or active TB, HIV positive, HIV viral load and CD4 count. All people with cough of any duration, fever, short breathing, sore throat, weight loss, hemoptysis, night sweat, arthralgia or myalgia should be investigated for TB. The Xpert MTB/RIF assay coupled with COVID-19 IgG/IgM should be indicated. A recent study has found that the specificities of serum IgM and IgG to diagnose COVID-19 were both more than 90% when compared to molecular detection [58]. If the Xpert MTB/RIF assay is negative, see options 2 and 3.

### Clinical management

#### Drug-drug interactions and clinical considerations

In the case of concurrent HIV and tuberculosis infection plus SARS-CoV-2 infection, the additional drug might cause interaction complicating the integrated therapy. In fact, some pharmaceutical interventions found for COVID-19 treatment including Protease inhibitors (PIs) (atazanavir, lopinavir, ritonavir, daranavir, raltegravir,cobicistat), remdesivir, ribavirin, arbidol, chloroquine, hydroxychloroquine, methylprednisolone, dexamethasone, anticoagulants and carrimycin may interfere and interact with TB and/or HIV treatments in multiple ways. Although protease inhibitors (PIs) were developed to be selective inhibitors of HIV-1 replication, they have shown inhibitory activity against a wide variety of pathogens [58], including SARS-CoV. Lopinavir / ritonavir (LPV/r) has a moderate anti-SARS-CoV-2 antiviral activity which works against the 3CL protease virus [59, 60]. A recent systematic review concluded that it is unclear whether LPV/r and other ART enhance clinical outcomes in severe symptomatic disease or prevent infection in patients at high- risk of COVID-19 based on the evidence available [61], as most of the studies included were case studies and also observational studies were low of power. Drug-drug interactions between PIs and rifampicin are known in HIV/TB co-infection. Studies have demonstrated that co-administration of PIs with rifampicin reduces PIs systemic concentration to less than 75% (cytochrome P 450 induction) [62, 63]. This may compromise COVID-19 treatment. Remdesivir should also not associate to rifampicin in COVID-19/TB co-infection because of strong induction [64]. A recent review has reported that chloroquine phosphate and hydroxychloroquine showed favorable outcomes in the recovery of COVID-19 patients [26, 65–68]. Both chloroquine and hydroxychloroquine are metabolized by hepatic cytochrome P450 enzyme 2D6 (CYP2D6) [69]. The most frequently involved in drug interactions are CYP3A4 and CYP2D6 [70]. The reduction in the efficacy of chloroquine when administered in conjunction with rifampicin may be due to the inducing effect of rifampicin on multidrug resistance associated protein (MRP) and development of CYP450 [70]. Additionally, high-dose chloroquine is more toxic than lower dose [64]. This is why; studies should clarify chloroquine and hydroxychloroquine dose adjustment in COVID-19/TB co-infection. Based on the above, dose adjustments should be taken into consideration in case PIs, chloroquine, hydroxychloroquine and remdesivir are administered with rifampicin. Another option is to shift rifampicin to rifabutin or adapted TB regimens without rifampicin. In contrast, clofazimine used in MDR-TB is a strong inhibitor of PIs, known substrates [71]. Then, caution should be taken when administered with PIs. Another TB drug with in vitro effect used in COVID-19 is carrimycin. Its use in COVID-19 may mitigate active TB and biases the TB diagnostic.

A study showed an association between corticosteroid use and lower mortality in COVID-19 patients [68]. Using a glucocorticoid in the early stages of the prognosis for a brief period of time could minimize the inflammation, but longer-term use could result in the risk of HIV and/or TB activation and even lack of treatment with TB. Careful use of corticosteroids with low-to-moderate doses in short courses is advised [68]. Besides, fibrosis and extensive pulmonary pathology secondary to TB and COVID-19, as defined in the introduction, can reduce drug penetration at the lung sites. It is a significant risk factor for bad TB outcomes in the event of potential infection or reactivation of TB [72]. This may also induce MDR-TB or extensively drug-resistant tuberculosis (XDR-TB) or recurrent pneumonia. Then, special considerations should be taken into account in the clinical management of COVID-19/TB lung fibrosis. Some RCTs are currently underway evaluating the safety and effectiveness of antifibrotic therapies on COVID-19 lung fibrosis [46].

Besides, liver and kidneys toxicities related to severe and critical COVID-19 need a tailored therapeutic approaches in HIV/TB co-morbidities due to some hepatotoxicity and nephrotoxicity of some HIV/TB drugs such as streptomycin, isoniazid, rifampicin, pyrazinamide, tenofovir disoproxil, atazanavir/ritonavir, lopinavir/ritonavir as well as HIV induced nephropathy and hepatitis associated to HIV.

#### Clinical management approach

1. Mild to Moderate COVID-19 associated with HIV/TB co-infection: Hospitalized in a special unit named COVID-19/TB units as risk patients. Start COVID-19 antiviral drugs, start or continue anti TB drugs according to the national guidelines and continue ART. Preferred COVID-19 antivirals are oseltamivir, chloroquine or hydroxychloroquine associated to LV/r or darunavir/cobicistat and Azithromycin may be indicated [68]. Chloroquine: 1 g PO once on Day 1, then 500 mg PO once daily for 4–7 days, hydroxychloroquine: 800 mg PO once on Day 1, then 400 mg PO once daily for 4–7 days [64] or lopinavir 400 mg/ritonavir 100 mg PO twice [65]. All of them should be associated with Azithromycin [64]. Drugs interactions should be reviewed as described above. Initial evaluation includes a chest x-ray, complete blood count (CBC), liver transaminases, renal function, inflammatory markers such as C-reactive protein (CRP), D-dimer, and ferritin, while not part of standard care, may have prognostic value.

2. Severe COVID-19 associated to HIV/TB co-infection: Hospitalized in COVID-19/TB unit as high-risk patients. Drug therapy and ventilator support are milestones. Clinicians can refer to COVID-19 antiviral therapy and immune-based therapy [64]. Start COVID-19 antiviral drugs as described in mild to moderate COVID-19, add immune-based therapy, initiate or continue anti TB drugs according to national guidelines and nephrotoxic ART regimens may be discontinued, switched to another ART regimen or adjusted dose based on the kidney function and drug-drug interactions [73]. Remdesivir is recommended in severe/critical COVID-19 however this cannot be administered with rifampicin [64]. Short period low-dose corticosteroid therapy is preferred over no corticosteroid therapy in HIV/TB co-infection and also the patients are in the intensive care unit [64]. Anticoagulant therapy mainly with low molecular weight heparin should be initiated early as this appears to be associated with better prognosis in severe COVID-19 patients [74]. Ventilator support, oxygen through a face mask and symptomatic therapy should be indicated. Initial evaluation includes chest x-ray/CT-scan and CBC should be indicated. Liver transaminases and renal function should be monitored regularly in consideration of COVID-19/HIV/TB drug-drug interactions and clinical considerations. Measurements of inflammatory markers, D-dimer, and ferritin are part of the management.

3. Critical COVID-19 associated to HIV/TB co-infection: Hospitalized in COVID-19/TB unit with ICU as high-risk patients. Infection control and testing, ventilator support, hemodynamic, and drug therapy are milestones [65]. Apply COVID-19, TB and HIV management as described in severe COVID-19. Short period low-dose corticosteroid therapy, anticoagulant therapy and norepinephrine as the first-choice vasopressor are recommended [64]. Anticoagulant therapy mainly with low molecular weight heparin appears to be associated with better prognosis in severe/critical COVID-19 patients with markedly elevated D-dimer [74]. There is strong evidence against the use of hydroxyethyl starches for the acute reanimation of adults with COVID-19 in shock [75]. In adults with COVID-19 in shock, if the peripheral oxygen saturation (SpO2) is < 92%, the review suggested starting supplemental oxygen if SpO2 is < 90% [75]. Initial evaluation includes chest x-ray/CT-scan and CBC should be indicated. Liver transaminases and renal function should be monitored regularly in consideration of COVID-19-HIV and TB drug-drug interactions and clinical considerations. Inflammatory markers, D-dimer, cardiac enzymes and ferritin monitoring should be part of the management.

4. Previous history of COVID-19 in HIV/TB co-infection: This group of cases should be treated as HIV/TB co-infection as described in different national guidelines. Therefore, emphasis should be put on the risk of severe lung fibrosis that may induce MDR-TB or XDR-TB. Ongoing trials are evaluating the safety and effectiveness of antifibrotic therapy in COVID-19 severe and critical patients [46]. This could be beneficial in COVID-19-HIV and TB co-infected cases due to their synergic roles in inducing pulmonary fibrosis.

## Conclusion

This is the first systematic review of the burden of COVID-19-HIV and TB co-infection in high burden HIV/TB countries. This review highlighted special considerations that should be taken in high burden HIV and TB countries at present and in the future. The results of the present descriptive analysis and meta-analysis of twenty one studies among which two were four case reports, eight case series, one case-control and eight cohort studies. Descriptive analysis has shown that SARS-CoV, MERS-CoV and COVID-19 associated with HIV/TB or TB are more common in males and the time-to-recovery is long compared to the non-exposure groups. Meta-analysis suggests that HIV/TB co-infection or TB exposures increase the risk of severe/critical COVID-19 and the mortality. The current TB group has an increased risk of COVID-19 compared to the previous TB. Additionally, the HIV/TB co-infected group has the highest risk in the COVID-19 mortality rate and poor recovery rate. This evidence is strong enough as substantial heterogeneities were absent in all the results (*I*^2^ values were less than 50% in all the meta-analysis).

Based on the results, the review offers special attention on diagnostics and management of COVID-19/HIV/TB and COVID-19/TB co-infections. TB diagnostic suggests four algorithms fast-tracking TB investigations in COVID-19/HIV/TB and COVID-19/TB co-infections. Well-structured clinical management has been suggested, focusing on COVID-19, HIV and TB drug-drug interactions and also COVID-19 clinical considerations.

Knowing that COVID-19 and TB may induce the development of severe lung disease leading to pulmonary fibrosis in the future, further studies are needed with cohorts of HIV/COVID-19 co-infected individuals. More research is needed to explore the effect of lung fibrosis related to COVID-19 in high burden HIV/TB countries. This pressing priority will shed light on the utility of prophylaxis treatments in preventing post-COVID-19 related LRTIs in high burden HIV/TB countries.

## Supplementary information

**Additional file 1: Table 1.** Quality assessment of included studies

## Data Availability

All data and material are presented in this review.

## Abbreviations

COVID-19: Coronavirus Disease 19
DAD: Diffuse Alveolar Damage
ICU: Intensive Care Unit
IGRA: Interferon Gamma Release Assay
HIV: Human Immunodeficiency Virus
LRTIs: Lower Respiratory Tract Infections
MERS-CoV: Middle East respiratory syndrome coronavirus
MDR-TB: Multidrug-resistant TB
NIH: National Institute of Health
NOS: Newcastle-Ottawa-Scale
NRTI: Nucleoside Reverse Transcriptase Inhibitor
PRISMA: Preferred Reporting Items for Systematic Reviews and Meta-Analysis
PLWH: People Living With HIV
PTB: Pulmonary TB
RIF: Rifampicin
RT-PCR: Real-time polymerase chain reaction
SARS-CoV: Severe acute respiratory syndrome coronavirus
TB: Tuberculosis
UNAIDS: The joint United Nations Programme on HIV/AIDS
USAID: U.S. Agency for International Development
WHO: World Health Organization
XDR-TB: Extensively drug-resistant tuberculosis
