# Supplementary material for: Implications of COVID-19 in high burden countries for HIV/TB: A systematic review of evidence
Source: BMC Infect Dis. 2020 Oct 9;20:744. doi: 10.1186/s12879-020-05450-4 (PMC7545798; doi:10.1186/s12879-020-05450-4)
Supplement: Supplementary file 1 — Additional file 1: Table 1. Quality assessment of included studies [file 12879_2020_5450_MOESM1_ESM.docx]

**Supplementary material: Quality assessment of included studies**

| Table 3.Quality assessment of included studies. | | | | | |
| --- | --- | --- | --- | --- | --- |
|  |  | **Quality assessment criteria** | | | |
| **Author (year)** | **Study design** | **Selection** | **Comparability** | **Outcome/ exposure** | **Overall quality** |
|  |  |  |  |  |  |
| Davies 2020 | Cohort | **** | * | *** | 8 |
|  |  |  |  |  |  |
| Liu 2020 | Case-control | ** | * | ** | 5 |
|  |  |  |  |  |  |
| Zhang 2020 | Cohort | ** | * | ** | 5 |
|  |  |  |  |  |  |
| Karla 2020 | Cohort | *** | * | *** | 7 |
|  |  |  |  |  |  |
| Du 2020 | Cohort | ** | * | *** | 6 |
|  |  |  |  |  |  |
| Li 2020 | Cohort | ** | * | *** | 6 |
|  |  |  |  |  |  |
| Zhang 2020b | Cohort | ** | * | *** | 6 |

Newcastle-Ottawa Scale was obtained to assess the selection, comparability and exposure of the case-control study, while the selection, comparability and outcome for the cohort study. -: no point; *: one point; **: two points; ***: three points; ****: four points.
